# Hyperbaric Oxygen Therapy for Suburethral Vaginal Mucosal Necrosis after Interstitial Irradiation for Recurrent Cervical Cancer

**DOI:** 10.1155/2021/1737975

**Published:** 2021-09-09

**Authors:** Yoshihisa Arakaki, Yuko Shimoji, Tadaharu Nakasone, Yusuke Taira, Tomoko Nakamoto, Wataru Kudaka, Keiko Mekaru, Yoichi Aoki

**Affiliations:** Department of Obstetrics and Gynecology, Graduate School of Medicine, University of the Ryukyus, 207 Uehara Nishihara Okinawa 903-0215, Japan

## Abstract

Patients with gynecological malignancies can develop radiation injuries, such as cystitis, proctitis, and soft tissue necrosis which have approved indications for hyperbaric oxygen therapy (HBOT). A 76-year-old Japanese woman with vaginal recurrence of cervical cancer was treated with the high-dose rate interstitial brachytherapy. Twenty-one months after the irradiation, she developed radiation necrosis on the external urethral opening. Two cycles of HBOT were performed. HBOT consisted of delivering 100% oxygen for 60 minutes at 2.4 atmospheres absolute. Pressure exposure was performed once daily, 5 days a week for 6 weeks. Eventually, the necrotic mucosa was completely replaced by the normal mucosa. No adverse effects were observed. We successfully treated a case of late adverse events of radiation therapy with HBOT. It was noninvasive and appears to be a useful treatment option which should be considered standard treatment practice.

## 1. Introduction

Radiation is a well-established therapy for the treatment of gynecological cancer, in particular primary and recurrent cervical cancer. However, radiation-related complications like delayed radiation injuries, in which histology shows hypovascular, hypoxic, and hypocellular tissues [1], may develop. Difficulty in treatment is often experienced as a unique characteristic of radiation therapy. Hyperbaric oxygen therapy (HBOT) has been used as a treatment modality to stimulate repair of radiation-induced vascular damage. It increases the oxygen gradient in damaged and radiated tissues, providing a stimulus for angiogenesis, and has been shown to be effective in treating radiation injuries that affect angiogenesis and potentially alter the underlying mechanism of tissue damage [2]. The Tenth European Consensus Conference on Hyperbaric Medicine includes HBOT for postradiation injury of soft tissues [3]. Patients with gynecological malignancies can develop radiation injuries, such as cystitis, proctitis, and soft tissue necrosis which have approved indications for HBOT in Japan [4]. Allen et al. demonstrated that there was a high level evidence for the use of HBOT for radiation proctitis, radiation cystitis, and necrosis in patients with gynecologic malignancies treated with radiation [5].

We report a case of successful treatment with HBOT for suburethral vaginal mucosal necrosis as a late adverse event of radiation therapy.

## 2. Case Report

**Figure 1:**
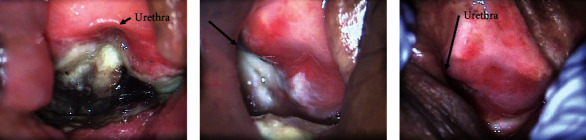
(a) A necrotic penetrating mucosa was observed in the suburethral vaginal wall. (b) The necrotic mucosa closed partially after 30 hyperbaric oxygen therapy (HBOT) sessions. (c) The necrotic mucosa was completely replaced by the normal mucosa after 60 HBOT sessions.

A 76-year-old Japanese woman underwent radical hysterectomy for cervical cancer stage IB1 on April 28, 2016. Eight months later, vaginal recurrence, 1 cm from the vaginal opening at 2 o'clock of the vaginal mucosa, occurred and the recurrent tumor was removed. However, 5 months after the recurrent tumor was removed, vaginal recurrence at the same site occurred. The high-dose rate interstitial brachytherapy (HDR-ISBT) was prescribed with a total dose of 42 Gray (Gy)/7 fractions for 4 days. HDR-ISBT treatment consisted of twice-daily irradiation of six Gy each time, with at least a 6-hour interval to provide the total prescribed dose. Twenty-one months after the irradiation, the patient presented with complaints of local pain and pain on urination. The necrotic mucosa was found on the posterior wall of the external urethral opening (Figure 1(a)). The radiologists confirmed that this was consistent with the site where interstitial irradiation was performed. No recurrent findings were observed on biopsy and magnetic resonance imaging. Mucosal necrosis by radiation late effects was diagnosed, and the patient was referred to the Department of Urology. Urethral repair was not indicated, and cystostomy was suggested. The patient did not wish to have the procedure. Accordingly, HBOT was preferred and started in October 2018. HBOT consisted of delivering 100% oxygen for 60 minutes at 2.4 atmospheres absolute. Pressure exposure was performed once daily, 5 days a week for 6 weeks. Nonsteroidal anti-inflammatory drugs (NSAIDs) were started for pain control. However, due to inadequacy of the NSAIDs, oral oxycodone was started. No significant change was observed in the mucosal necrosis after 30 sessions of HBOT (Figure 1(b)). In January 2019, the patient complained of worsening pain due to the local infection in the mucosa necrosis. Hence, intravenous 1 g/day of cefmetazole, which is effective for urinary tract infection and gynecological infection, and 14.4 mg subcutaneous injection of oxycodone were started, and the pain and inflammatory findings improved. The second course of HBOT with the same prescription was resumed from March 2019 to April 2019. As pain improved during this period, oxycodone medication was discontinued. No adverse effects were observed. Eventually, the necrotic mucosa was completely replaced by the normal mucosa (Figure 1(c)).

## 3. Discussion

We successfully treated late adverse events of radiation necrosis with hyperbaric oxygen therapy. The mechanism by which HBOT is effective for radiation injuries is as follows: the amount of dissolved oxygen in plasma becomes 10–20 times higher than normal, increasing the rate of oxygen diffusion into tissues. Normally, the vascular regulatory function causes vasoconstriction as a biological response to prevent cell death due to oxygen toxicity, thereby controlling the amount of oxygen supplied. However, in damaged tissues under hypoxic conditions such as ischemic tissues, vasoconstriction does not occur and fibroblasts are activated, improving the balance between collagen synthesis and angiogenesis and promoting wound healing [2, 6, 7].

The incidence of delayed radiation injury had been reported to be 23% in gynecologic malignancies [5]. HBOT has been used successfully for years in treating head and neck radiation necrosis (radionecrosis). Marx reported a controlled, nonrandomized study of soft tissue radionecrosis of the neck. He showed fewer infections in the HBOT group, less dehiscence, and improved healing times [1]. However, there is an absence of randomized controlled trials that studied the effects of HBOT on radiation necrosis in gynecologic cancers. Most of the studies were retrospective; however, they showed excellent results, often resolving necrotic wounds and fistulas [8]. Furthermore, a review of literature by Allen et al. recommended that HBOT should be considered for the treatment of radiation necrosis, and it is reasonable to apply the same standards used in gynecologic malignancies [5]. The recommendations they have made are limited by the relative paucity of studies on HBOT in gynecologic malignancies. This could be due to the difficulty in conducting large studies in hyperbaric chambers. Serious adverse events due to HBOT were rare, while most of adverse events were minor and self-limiting [9].

We successfully treated a case of late adverse events of radiation therapy with HBOT. It was noninvasive and appears to be a useful treatment option which should be considered standard treatment practice.
